# Diminishing Loss Sensitivity during Risky Decision-Making among Individuals with Gambling Disorder

**DOI:** 10.1192/j.eurpsy.2026.10501

**Published:** 2026-07-31

**Authors:** H. Wei, G. Zhong, J. Liu, Y. Wei, X. Zhang, P. Yang, M. Zhao, J. Du

**Affiliations:** 1 Shanghai Mental Health Center, Shanghai Jiao Tong University School of Medicine; 2 Shanghai Key Laboratory of Psychotic Disorders, Shanghai Mental Health Center, Shanghai, China

## Abstract

**Introduction:**

Gambling disorder (GD) poses severe impacts on both individuals and society. Impairment in risky decision-making is a key behavioral characteristic of GD, but the underlying cognitive processes of these deficits remain unclear.

**Objectives:**

This study decomposed the risky decision-making processes of GD with cognitive computational modeling.

**Methods:**

A total of 100 participants with GD and 59 healthy controls (HCs) were recruited to complete psychometric assessments and the Balloon Analog Risk Task. Since GD involved abnormal loss evaluation in decision-making, we developed a novel cognitive model incorporating diminishing loss sensitivity and revealed the processes underlying the risk-taking behaviors with hierarchical Bayesian analysis.

**Results:**

GD participants exhibited stronger loss aversion (*p* < 0.001) but faster-diminishing loss sensitivity (*p* < 0.001), regardless of severity, which drove the deficits in the overall performance of risky decision-making (*p* < 0.001) (Fig1). Overconfident prior belief (*p* = 0.002) and higher updating rate (*p* < 0.001) were observed among participants with GD (Fig3). Diminishing loss sensitivity was negatively correlated with impulsivity (*p* = 0.021), and loss aversion was negatively related to craving for gambling (*p* = 0.027) (Fig4).

**Table 1.** Demographic and clinical characteristics of participants Table 1. Long description.

Characteristic	Total, N = 159	HC, n = 59	GD, n = 100	*F*/*H*	*p*	Effect Size
Age, Years	30.84 ± 7.91	31.71 ± 9.91	30.33 ± 6.45	*H*_1_ = 0.28	0.595	*η^2^* = 0
Education, Years	14.96 ± 3.25	15.27 ± 4.49	14.77 ± 2.23	*H*_1_ = 2.57	0.109	*η^2^* = 0.010
BIS Score	82.78 ± 17.64	69.20 ± 16.04	90.79 ± 13.09	*F*_1, 157_ = 85.127	< 0.001	*η^2^* = 0.352
VAS Score			3.65 ± 3.34			

HC: Health controls; GD: Gambling disorder; BIS: Barret Impulsivity Scale; VAS: Visual Analog Scale for gambling craving.

**Table 2.** Demographic and clinical characteristics of subgroups of participants. Table 2. Long description.

Characteristic	Total, N = 159	HC, n = 59	Mild&Moderate, n = 46	Severe, n = 54	*F*/*H*	*p*	Effect Size
Age, Years	30.84 ± 7.91	31.71 ± 9.91	30.78 ± 5.86	29.94 ± 6.95	*H*_2_ = 1.48	0.476	*η^2^* = 0
Education, Years	14.96 ± 3.25	15.27 ± 4.49	14.93 ± 1.84	14.63 ± 2.52	*H*_2_ = 2.60	0.273	*η^2^* = 0.004
BIS Score	82.78 ± 17.64	69.20 ± 16.04	88.50 ± 13.99	92.74 ± 12.07	*F*_2, 156_ = 44.001	< 0.001	*η^2^* = 0.361
VAS Score			3.07 ± 3.01	4.15 ± 3.55	*H*_1_ = 2.00	0.158	*η^2^* = 0.010

HC: Health controls; Mild&Moderate: Mild and moderate gambling disorder participants; Severe: Severe gambling disorder participants; BIS: Barret Impulsivity Scale; VAS: Visual Analog Scale for gambling craving.

**Image 1:**
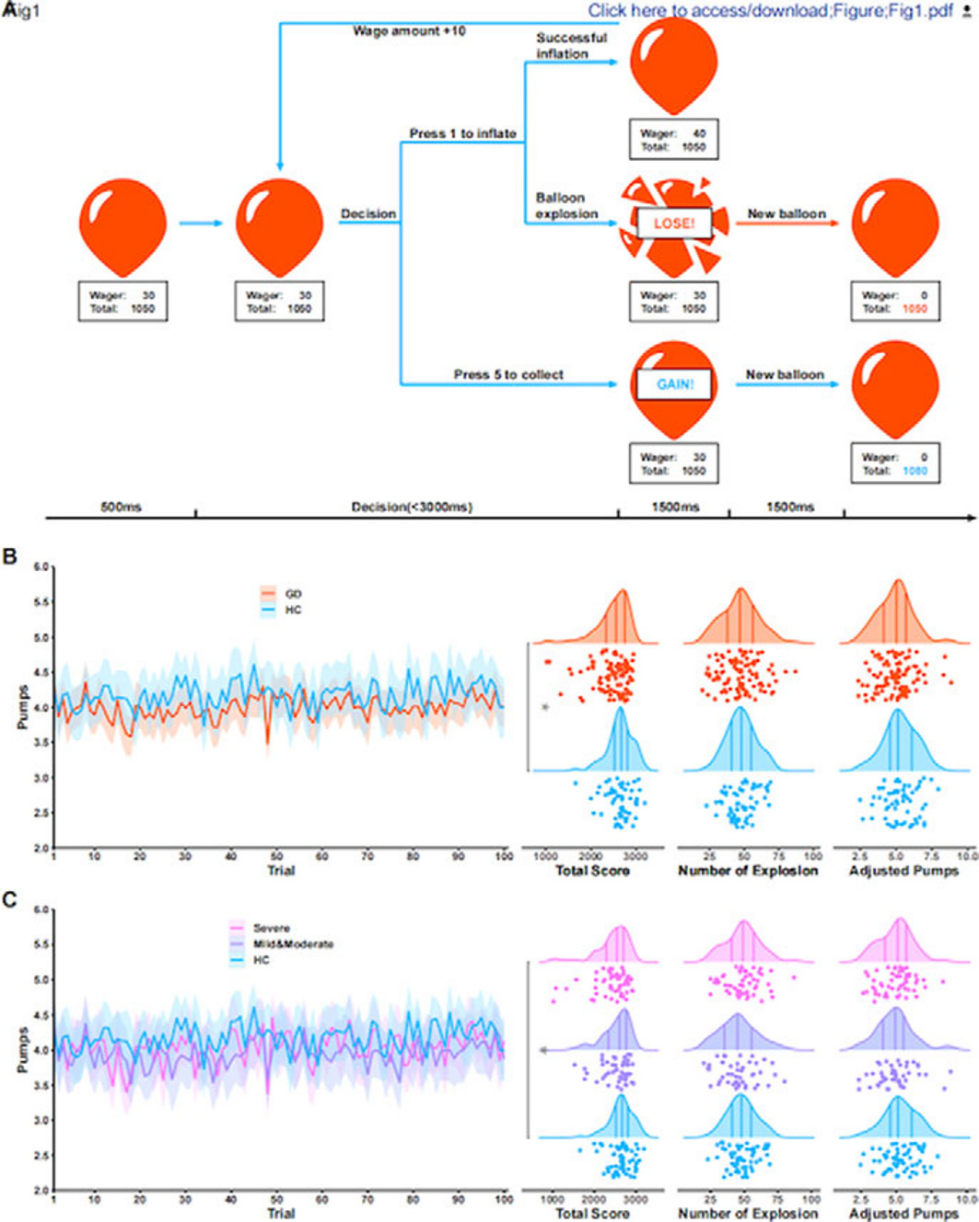
Image 1: Long description.

**Image 2:**
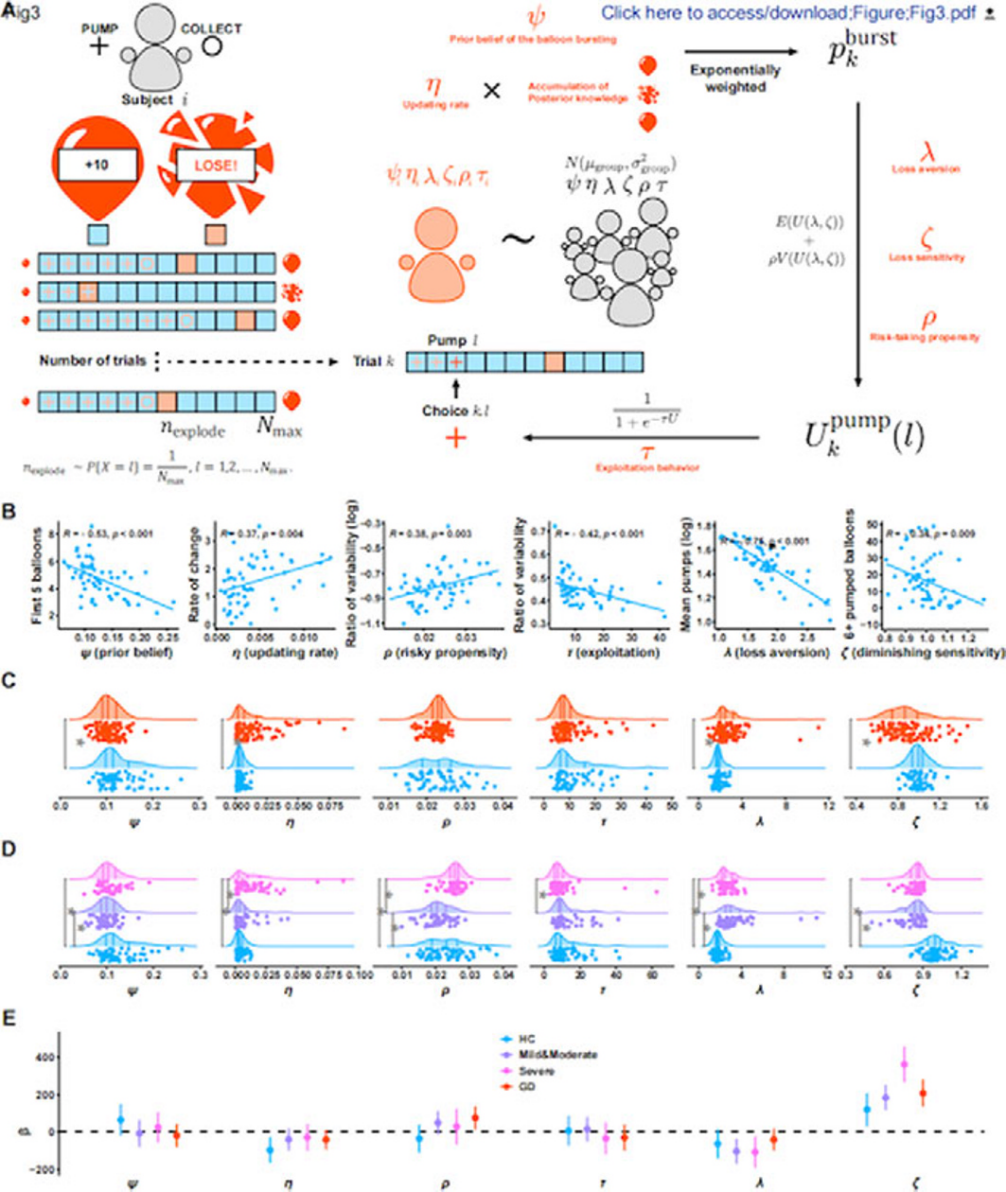
Image 2: Long description.

**Image 3:**
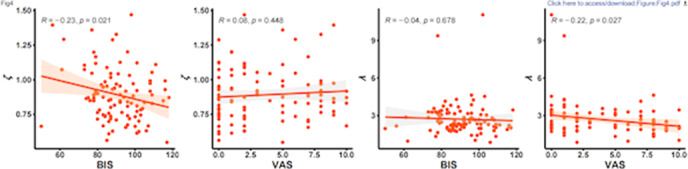
Image 3: Long description.

**Conclusions:**

This research provides novel perspectives on the cognitive processes underlying the risky decision-making of GD patients, highlighting the role of diminishing loss sensitivity during loss evaluation and its clinical implications, which inspires future research on assessment and psychotherapy for GD.

**Disclosure of Interest:**

None Declared

## Long descriptions

### Table 1. Long description

The table consists of seven columns: Characteristic, Total (N = 159), H C (n = 59), G D (n = 100), F or H, p, and Effect Size.

* Age, Years: Total 30.84 plus or minus 7.91; H C 31.71 plus or minus 9.91; G D 30.33 plus or minus 6.45; H sub 1 = 0.28; p = 0.595; Effect Size eta-squared = 0.

* Education, Years: Total 14.96 plus or minus 3.25; H C 15.27 plus or minus 4.49; G D 14.77 plus or minus 2.23; H sub 1 = 2.57; p = 0.109; Effect Size eta-squared = 0.010.

* B I S Score: Total 82.78 plus or minus 17.64; H C 69.20 plus or minus 16.04; G D 90.79 plus or minus 13.09; F sub 1, 157 = 85.127; p < 0.001; Effect Size eta-squared = 0.352.

* V A S Score: G D 3.65 plus or minus 3.34 (other columns blank).

Abbreviations: H C stands for Health controls; G D stands for Gambling disorder; B I S stands for Barret Impulsivity Scale; V A S stands for Visual Analog Scale for gambling craving.

Navigate back to Table 1..

### Table 2. Long description

The table consists of 8 columns: Characteristic, Total (N = 159), H C (n = 59), Mild and Moderate (n = 46), Severe (n = 54), F or H statistic, p-value, and Effect Size (eta-squared).

* Age, Years: Total 30.84 plus or minus 7.91; H C 31.71 plus or minus 9.91; Mild and Moderate 30.78 plus or minus 5.86; Severe 29.94 plus or minus 6.95. H sub 2 = 1.48, p = 0.476, eta-squared = 0.

* Education, Years: Total 14.96 plus or minus 3.25; H C 15.27 plus or minus 4.49; Mild and Moderate 14.93 plus or minus 1.84; Severe 14.63 plus or minus 2.52. H sub 2 = 2.60, p = 0.273, eta-squared = 0.004.

* B I S Score: Total 82.78 plus or minus 17.64; H C 69.20 plus or minus 16.04; Mild and Moderate 88.50 plus or minus 13.99; Severe 92.74 plus or minus 12.07. F sub 2, 156 = 44.001, p < 0.001, eta-squared = 0.361.

* V A S Score: Mild and Moderate 3.07 plus or minus 3.01; Severe 4.15 plus or minus 3.55. H sub 1 = 2.00, p = 0.158, eta-squared = 0.010.

Abbreviations: H C stands for Healthy Controls; B I S stands for Barratt Impulsivity Scale; V A S stands for Visual Analog Scale for gambling craving.

Navigate back to Table 2..

### Image 1: Long description

Panel A is a flowchart of a balloon task. It begins on the left with a balloon at 500 m s. A decision phase follows where pressing 1 inflates the balloon, increasing the wager by 10. This leads to either successful inflation or a balloon explosion labeled LOSE. Alternatively, pressing 5 collects the current wager, labeled GAIN. Both outcomes lead to a new balloon after 1500 m s.

Panel B shows behavioral data for G D in orange and H C in blue. A line graph on the left plots Pumps from 2.0 to 6.0 over 100 trials, showing fluctuating but stable trends around 4.0. To the right are three columns of distribution plots for Total Score, Number of Explosion, and Adjusted Pumps. G D shows slightly higher density in higher pump counts compared to H C.

Panel C displays the same metrics for three groups: Severe in pink, Mild and Moderate in purple, and H C in blue. The line graph shows overlapping trends across 100 trials. The distribution plots on the right show that the Severe group has a wider distribution and higher peaks in the Adjusted Pumps and Number of Explosion categories compared to the H C group.

Navigate back to Image 1:.

### Image 2: Long description

Panel A: A flowchart titled Subject i shows a balloon task where pumping adds 10 points but bursting causes a loss. Mathematical variables include eta for updating rate, psi for prior belief, and tau for exploitation behavior. A formula at the bottom defines n sub explode as a probability function.

Panel B: Six scatter plots with linear regression lines showing correlations between behavioral metrics and model parameters.

- First 5 balloons versus psi shows a negative correlation (R = -0.53).

- Rate of change versus eta shows a positive correlation (R = 0.37).

- Ratio of variability (log) versus rho shows a positive correlation (R = 0.38).

- Ratio of variability versus tau shows a negative correlation (R = -0.42).

- Mean pumps (log) versus lambda shows a negative correlation (R = -0.76).

- 6 plus pumped balloons versus zeta shows a negative correlation (R = -0.34).

Panel C and D: Raincloud plots showing the distribution of parameters psi, eta, rho, tau, lambda, and zeta across different groups, with individual data points below density curves.

Panel E: A coefficient plot showing beta values for the six parameters across four groups: H C (blue), Mild and Moderate (purple), Severe (pink), and G D (red). The y-axis ranges from -200 to 400. Most parameters hover near the zero baseline except for zeta, which shows a significant positive beta value across all groups.

Navigate back to Image 2:.

### Image 3: Long description

A four-panel scatter plot series. Each plot contains red data points, a solid red regression line, and a light orange shaded confidence interval.

Panel 1 (Far Left)

- X-axis: B I S ranging from 60 to 120.

- Y-axis: zeta ranging from 0.75 to 1.50.

- Statistics: R equals negative 0.23, p equals 0.021.

- Trend: A moderate negative linear correlation.

Panel 2 (Middle Left)

- X-axis: V A S ranging from 0.0 to 10.0.

- Y-axis: zeta ranging from 0.75 to 1.50.

- Statistics: R equals 0.08, p equals 0.448.

- Trend: A near-flat, slightly positive linear correlation.

Panel 3 (Middle Right)

- X-axis: B I S ranging from 60 to 120.

- Y-axis: lambda ranging from 3 to 9.

- Statistics: R equals negative 0.04, p equals 0.678.

- Trend: A near-flat, slightly negative linear correlation.

Panel 4 (Far Right)

- X-axis: V A S ranging from 0.0 to 10.0.

- Y-axis: lambda ranging from 3 to 9.

- Statistics: R equals negative 0.22, p equals 0.027.

- Trend: A moderate negative linear correlation.

Navigate back to Image 3:.

